# Salvage PRRT with ^177^Lu-DOTA-octreotate in extensively pretreated patients with metastatic neuroendocrine tumor (NET): dosimetry, toxicity, efficacy, and survival

**DOI:** 10.1186/s12885-019-6000-y

**Published:** 2019-08-08

**Authors:** S. Rudisile, A. Gosewisch, V. Wenter, M. Unterrainer, G. Böning, F. J. Gildehaus, W. P. Fendler, C. J. Auernhammer, C. Spitzweg, P. Bartenstein, A. Todica, H. Ilhan

**Affiliations:** 1 Department of Nuclear Medicine, University Hospital, LMU Munich, Munich, Germany; 2 Department of Internal Medicine 4, University Hospital, LMU Munich, Munich, Germany; 3 ENETS Centre of Excellence, Interdisciplinary Center of Neuroendocrine Tumors of the GastroEnteroPancreatic System (GEPNET-KUM), LMU Munich, Munich, Germany; 4 Department of Nuclear Medicine, University Hospital, University of Essen, Essen, Germany

**Keywords:** PRRT, Salvage therapy, NET, SPECT, Dosimetry, Survival

## Abstract

**Background:**

NETTER-1 trial demonstrated high efficacy and low toxicity of four cycles of Peptide Receptor Radionuclide Therapy (PRRT) in patients with metastasized NET. The present study evaluates the outcome of further PRRT cycles in the so called salvage setting in patients after initial response to four therapy cycles and later progression.

**Methods:**

Thirty five patients (pat.) (25 male, 10 female, 63 ± 9 years) with progressive, metastasized NET (23 small intestinal, 5 lung, 4 CUP, 1 rectal, 1 gastric and 1 paraganglioma) were included. All patients previously received 4 PRRT cycles with ^177^Lu-DOTATATE and showed initial response. SPECT based dosimetry was applied to determine kidney and tumor doses. Therapy response was evaluated using ^68^Ga-DOTATATE PET/CT (with high dose CT), CT alone or MRI (RECIST 1.1), toxicity was defined using CTCAE 5.0 criteria. ^99m^Tc99-MAG3 scintigraphy was used to assess potential renal tubular damage. Progression free survival (PFS) and Overall survival (OS) analysis was performed with the Kaplan-Meier-method.

**Results:**

The median PFS after initial PRRT was 33 months (95% CI: 30–36). The mean cumulative dose for including salvage PRRT was 44 GBq (range 33.5–47). One pat. (2.9%) showed grade 3 hematotoxicity. Kidney dosimetry revealed a mean cumulative kidney dose after a median of 6 PRRT cycles of 23.8 Gy. No grade 3 / 4 nephrotoxicity or relevant decrease in renal function was observed. Follow-up imaging was available in 32 patients after salvage therapy. Best response according to RECIST 1.1. was PR in one patient (3.1%), SD in 26 patients (81.3%) and PD in 5 patients (15.6%). PFS after salvage therapy was 6 months (95% CI: 0–16; 8 patients censored). Mean OS after initial PRRT was 105 months (95% CI: 92–119) and 51 months (95% CI: 41–61) after start of salvage therapy. Median OS was not reached within a follow-up of 71 months after initial PRRT and 25 months after start of salvage PRRT, respectively.

**Conclusions:**

Salvage therapy with ^177^Lu-DOTATATE is safe and effective even in patients with extensive previous multimodal therapies during disease progression and represents a feasible and valuable therapy option for progressive NET.

**Electronic supplementary material:**

The online version of this article (10.1186/s12885-019-6000-y) contains supplementary material, which is available to authorized users.

## Background

Neuroendocrine tumors (NET) are rare malignancies with an increasing age-adjusted incidence of 6–7 per 100.000 in 2012 compared to 1 per 100.000 in 1973 [1]. The highest increase in incidence is observed in localized and Grade I NET [1]. However, metastatic disease is observed in 20–40% of NET patients at initial diagnosis and has a significant impact on survival [2–4]. The over-expression of somatostatin receptors (SSTR) on the cell surface of NET [5] provides a theragnostic approach using radiolabeled somatostatin analogs such as ^68^Ga-DOTA-D-Phe-Tyr3-octreotate (^68^Ga-DOTATATE) for positron emission tomography / computed tomography (PET/CT) imaging and the β-emitter ^177^Lu-DOTA-D-Phe-Tyr3-octreotate (^177^Lu-DOTATATE) for peptide receptor radionuclide therapy (PRRT), which established as a safe and effective therapy option in metastatic, well-differentiated NET [6–8]. Recently, in the prospective Phase 3 trial NETTER-1 the combination of ^177^Lu-DOTATATE and 30 mg octreotoide LAR demonstrated longer PFS and OS in midgut NET patients compared to 60 mg octreotide LAR alone [9], which led to the approval of Lutathera® (^177^Lu-DOTA-D-Phe-Tyr3-octreotate) by the U.S. Food and Drug Administration (FDA) and the European Medicines Agency (EMA). Current guidelines recommend 3 to 5 cycles of ^177^Lu-DOTATATE with a standard dose of 7.4 GBq per cycle and an interval of 6–12 weeks between cycles [10, 11]. Even though ^177^Lu-DOTATATE demonstrated unprecedented survival benefit, metastases eventually recur with need for further treatment. Several studies indicate that additional PRRT cycles using ^177^Lu-DOTATATE in the so called salvage therapy setting are feasible, safe and effective [12–15].

Most prior salvage PRRT studies were performed in patients receiving ^177^Lu-DOTATATE salvage treatments without other therapy options between initial PRRT and re-challenge. However, given the broad variety of treatment options at tumor progression (e.g. biotherapy, surgery, chemotherapy, everolimus, antibody therapy and local ablative therapies such as radiofrequency ablation, transarterial chemoembolization and radioembolization [10]), it is of high interest to understand the outcome and side effects of salvage PRRT in patients within the multimodal treatment concept for metastatic NET. Hence, the aim of this retrospective single center study was to evaluate the toxicity, efficacy and survival after salvage PRRT in an extensively pretreated group of metastatic NET patients after treatment failure and and to identify possible parameters influencing survival. Due to a lack of published data for singe photon emission computed tomography (SPECT) based dosimetry after salvage ^177^Lu-DOTATATE therapy, SPECT image dosimetry of kidneys and metastases was performed for each therapy cycle.

## Methods

### Patients

Between August 2006 and May 2018 427 patients with well differentiated NET received at least one cycle of ^177^Lu-DOTATATE at the department of Nuclear Medicine, LMU Munich. Salvage PRRT, defined as a re-challenge with one or more ^177^Lu-DOTATATE therapy cycles after 4 initial PRRT cycles with initial response was performed in 56 patients. Patients receiving PRRT with ^90^Y labeled somatostatin analogs or ^177^Lu-DOTATATE therapy without SPECT based dosimetry were excluded from the analysis. A total of 35 consecutive patients (median 63 ± 9 years, range 45–81 years) were included in this retrospective analysis. All patients fulfilled the inclusion criteria for PRRT according to current guidelines [10, 11]. ^68^Ga-DOTATATE PET/CT was performed in each patient to determine sufficient uptake of somatostatin-analogs prior to PRRT. This retrospective analysis was performed in compliance with the principles of the Declaration of Helsinki and its subsequent amendments and was approved by the local Ethics Committee (approval number 19–027). The requirement to obtain informed consent was waived.

### ^177^Lu-DOTATATE treatment

Radiolabeling of ^177^Lu-DOTATATE was performed according to previously described protocols [16]. No-carrier added ^177^Lutetium was obtained from Isotope Technologies Garching GmbH (Garching, Germany), DOTA^0^,TYR^3^-octreotate was obtained from ABX advanced biochemical compounds (Dresden, Germany). ^177^Lu-DOTATATE was administrated with a standard dose of 7.4 GBq in intervals of 10 to 12 weeks (median: 11 weeks) between cycles 1 to 4 and in the salvage setting. Coinfusion of positively charged amino acids (2.5% Lysin and 2.5% Arginine) was started 30 min before each treatment for kidney protection. ^177^Lu-DOTATATE was injected i.v. within 10 min using a pump system.

### ^177^Lu-DOTATATE dosimetry

Dosimetry data were acquired for each therapy cycle. SPECT images were acquired on a dual-headed Symbia T2 SPECT/CT or on an E.CAM SPECT system (Siemens Medical Solutions, Erlangen, Germany) at 24, 48 and 72 h post injection over the complete abdomen with a scan time of 15 min according to previously described scan protocols [17, 18]. The co-registered diagnostic CT from the pre-therapeutically performed ^68^Ga-DOTATATE PET/CT scan was used for anatomical correlation-and attenuation correction -during quantitative SPECT reconstruction (rigid-body co-registration PMOD Version 3.609, PMOD Technologies, Zurich, Switzerland). For quantitative SPECT reconstruction a MAP algorithm with a penalty factor of 0.001, 20 iterations and 16 subsets was chosen, which further included scatter correction and resolution compensation (Hermes Hybrid Recon 1.4.2; Hermes Medical Solutions, Stockholm, Sweden). For estimation of absorbed dose values, volumes of interest (VOI) were subsequently delineated for kidneys and metastases, using an isocontour with a threshold of 30% (PMOD Version 3.609). As recently described, radiation absorbed doses were estimated by mono-exponentially time-activity-curve fitting [19]. According to the method of Garske et al.*,* the latest available time point in combination with the effective half-life of the previous cycle was used for absorbed dose estimation in case of less than three available time points [20]. Kidney volumes and masses were delineated and calculated from the pre-therapeutic diagnostic CT. For final absorbed dose estimation the time-integrated kidney or tumor activities were multiplied by mass-scaled S-values for either kidneys or tumors, as described previously [17, 21].

### Toxicity evaluation

For each PRRT cycle, laboratory analysis was performed 1 day prior to treatment, during the following inpatient stay and re-evaluated 2 and 6 weeks after each therapy. During follow-up laboratory parameters were re-evaluated every three months. Hematological parameters were determined with Common Terminology Criteria for Adverse Events (CTCAE) V5.0. Hematological and clinical parameters including WBC, erythrocytes, Hb, platelet count, cumulative absorbed kidney dose, Ki67, and post-therapeutical weight loss were determined and noted before and after each therapy cycle and during follow-up. Renal function was examined on the basis of glomerular filtration rate in plasma creatinine and the tubular extraction rate (TER) determined by ^99m^Tc MAG3 renal scintigraphy prior to each therapy cycle and during follow-up. Renal scintigraphy with ^99m^Tc-labelled MAG3 was performed on a doubled headed gamma camera (Siemens E.Cam; Siemens; Erlangen, Germany) equipped with a low-energy, high-resolution collimator according to previously described protocols [22]. The annual TER decrease (ml/min/1.73 m^2^) was normed to the lower limit as recently described by Werner et al. [23]. The annual decrease of the TER was determined by linear regression.

### Response assessment

During follow-up ^68^Ga-DOTATATE PET/CT (with high dose CT), diagnostic CT or MRI was performed in an interval of three months. Tumor response was assessed using the Response Evaluation Criteria in Solid Tumors 1.1 (RECIST 1.1) [24]. Disease control was defined as patients with a complete response (CR), partial response (PR), or stable disease (SD).

### Statistics

Statistical analysis was performed with the SPSS Software Package (SPSS Version 25; IBM). Statistical evaluations include overall and progression free survival analysis according to the Kaplan-Meier curve method, correlation analysis using Spearman rank correlation analysis, log-rank test and Pearson’s chi-squared Test. The assessment of the effect of multiple parameters (WBC, erythrocytes, Hb, platelet count, cumulative absorbed kidney dose, Ki67, sex, post-therapeutical weight loss, therapy response and age) on survival was performed with Kaplan-Meier and COX proportional hazard model (according to bivariate Variables). Median follow-up time and median OS were calculated from the date of the first PRRT cycle. PFS was calculated from the time of first PRRT until progression and from the start of salvage therapy. Differences between groups were described as significant at *p* < 0.05. Cut-off values were determined with the receiver operating characteristics (ROC) method. Data are presented as either mean or median values with standard deviation (SD) or standard error of the mean (SEM).

## Results

### Patient characteristics

Thirty five patients (25 male, 10 female; median 62 years, range 45–81 years) received at least one salvage PRRT cycle (median: 2) after 4 initial PRRT cycles. A total of 210 PRRT cycles with 70 cycles in the salvage setting have been performed with a cumulative median activity of 44 GBq (range: 33.5–47.0). Detailed information is given in Table 1. With a median of 39 months (range: 13–85 months) after 4 initial PRRT cycles, two patients received 1 cycle, 32 patients 2 cycles and one patient 4 cycles of salvage PRRT. Primary tumors were located in the small intestine (*n* = 23), lungs (*n* = 5), in the rectum (*n* = 1) and stomach (n = 1). Four patients had cancer of unknown primary (CUP) and one patient paraganglioma. Seven patients (20%) had a Ki-67 < 2% and 22 patients (62.9%) had a Ki-67 between 2 and 20%. At the time of PRRT, liver metastases were found in 31 patients (88.6%), lymph node and bone metastases in 12 (34.3%) patients, respectively, and peritoneal metastasis in 7 (14.3%).

**Table 1:** Patient characteristics

Number of patients	35
Sex	
Male (%)	25 (71.4)
Female (%)	10 (28.6)
Age (years) median range	62.5 (45–81)
Total cycles median (range)	6 (5–8)
Cumulative activity (GBq) median (range)	44 (33.5–47)
Primary tumor	
Small intestinal (%)	23 (65.7)
Lungs (%)	5 (14.3)
Cancer of unknown primary (%)	4 (11.4)
Rectal (%)	1 (2.9)
Gastric (%)	1 (2.9)
Paraganglioma (%)	1 (2.9)
Ki-67 proliferation index	
< 3%	7 (20.0)
3–20%	22 (62.9)
Not evaluable	6 (17.1)
Metastases (%)	
Liver (%)	31 (88.6)
Lymph nodes (%)	12 (34.3)
Bone (%)	12 (34.3)
Peritoneal (%)	7 (14.3)

### Previous therapies

Table 2 provides a summary of all applied therapies during disease progression prior to PRRT, between initial and start of salvage PRRT and after salvage PRRT. The vast majority of patients underwent resection prior to their first PRRT cycle (23 patients, 67.7%). Biotherapy was performed in 22 patients (62.9%), local ablative liver therapies in 12 patients (34.3%), chemotherapy in 2 (5.7%) and bone targeted therapies including bisphosphonates and denosumab in 7 patients (20.0%). During disease progression after initial PRRT and prior to salvage PRRT, surgery and local ablative liver therapies were performed in 9 patients (25.7%), respectively. Additional biotherapy and bone targeted therapies were performed in 5 patients (14.3%), respectively. One patients received everolimus, 1 patient antibody therapy prior to PRRT and 3 patients (8.6%) radiotherapy. Main additional therapies during disease progression after salvage PRRT included local ablative liver therapies in 3 patients (8.6%), chemotherapy in 7 (20.0%), everolimus in 10 (28.6%), antibody therapy in 8 (22.9%), bone targeted therapies in 4 patients (11.4%) and radiotherapy in 1 patient (2.9%).

**Table 2:** Applied treatment options during disease progression in all patients (*n* = 35)

	Therapies prior to initial PRRT	Additional therapies after initial PRRT and prior to salvage PRRT	Additional therapies after salvage PRRT
Surgery	23 (65.7%)	9 (25.7%)	–
Biotherapy	22 (62.9%)	5 (14.3%)	–
Local ablative therapy (RFA, TACE, SIRT)^a^	12 (34.3%)	9 (25.7%)	3 (8.6%)
Chemotherapy	2 (5.7%)	1 (2.9%)	7 (20.0%)
Everolimus	–	1 (2.9%)	10 (28.6%)
Protein Kinase Inhibitor / Antibody^b^	–	–	8 (22.9%)
Radiotherapy	2 (5.7%)	3 (8.6%)	1 (2.9%)
Bone targeted therapy^c^	7 (20.0%)	5 (14.3%)	4 (11.4%)

^a^*RFA* Radiofrequency ablation, *TACE* transarterial chemoembolisation, *SIRT* selective internal radiotherapy; ^b^ including tyrosine kinase inhibitors and bevacizumab, ^c^ Denosumab and Bisphosphonates

### Therapy associated hematotoxicity

One patient was lost to follow-up after salvage PRRT and excluded from the toxicity analysis. Only one patient (2.9%) showed grade 3 hematotoxicity after salvage PRRT according to CTCAE 5.0 criteria. Compared to mean baseline values prior to initial PRRT and prior to salvage PRRT, white blood cell (WBC) count decreased significantly after the initial four PRRT cycles (6.59 vs. 4.48, *p* < 0.001) and after salvage PRRT (6.62 vs. 5.08, p < 0.001). Likewise, erythrocyte and platelet count decreased after the initial four PRRT cycles and after salvage PRRT. Detailed numbers are given in Tables 3 and 4. Hematological parameters after initial four PRRT cycles recovered to baseline values comparable to values prior to initial PRRT.

**Table 3:** Hematological parameters before and after initial PRRT

	Baseline (mean ± SD)	n=	After 4 Cycles	Difference (%)	*p*-value (paired t-test)
WBC (10^3^/μl)	6.59 ± 2.12	35	4.48 ± 1.29	− 32.0	< 0.001
Erythrocytes (10^6^/μl)	4.48 ± 0.63	35	4.11 ± 0.54	−8.3	< 0.001
HB (g/dl)	13.43 ± 2.81	35	13.17 ± 1.40	− 1.9	= 0.506
PLT count (10^3^/μl)	254.86 ± 110.57	35	167.71 ± 50.62	−34.2	< 0.001

**Table 4:** Hematological parameters before and after salvage PRRT

	Baseline (mean ± SD)	n=	After Salvage PRRT	Difference (%)	*p*-value (paired t-test)
WBC (10^3^/μl)	6.62 ± 2.14	34	5.08 ± 2.01	−23.3	= 0.001
Erythrocytes (10^6^/μl)	4.49 ± 0.64	34	3.89 ± 0.70	−13.4	< 0.001
HB (g/dl)	13.44 ± 2.06	34	12.30 ± 2.06	−8.5	= 0.21
PLT count (10^3^/μl)	258.62 ± 109.94	34	200.71 ± 93.761	−22.4	= 0.016

### Therapy associated nephrotoxicity

No patient showed grade 3 or grade 4 nephrotoxicity according to CTCAE 5.0 during or after treatment. The absolute annual decrease in TER was 8 ± 12 ml/min/1.73 m^2^ resulting in an annual TER decrease of 0.03 ± 0.07 in linear regression analysis (Additional file 1: Table S5). This represents an annual decrease of 2.25 ± 0.48% compared to baseline values and normed to the age specific lower TER limit.

### SPECT based tumor and kidney dosimetry during PRRT and salvage PRRT

**Fig. 1:**
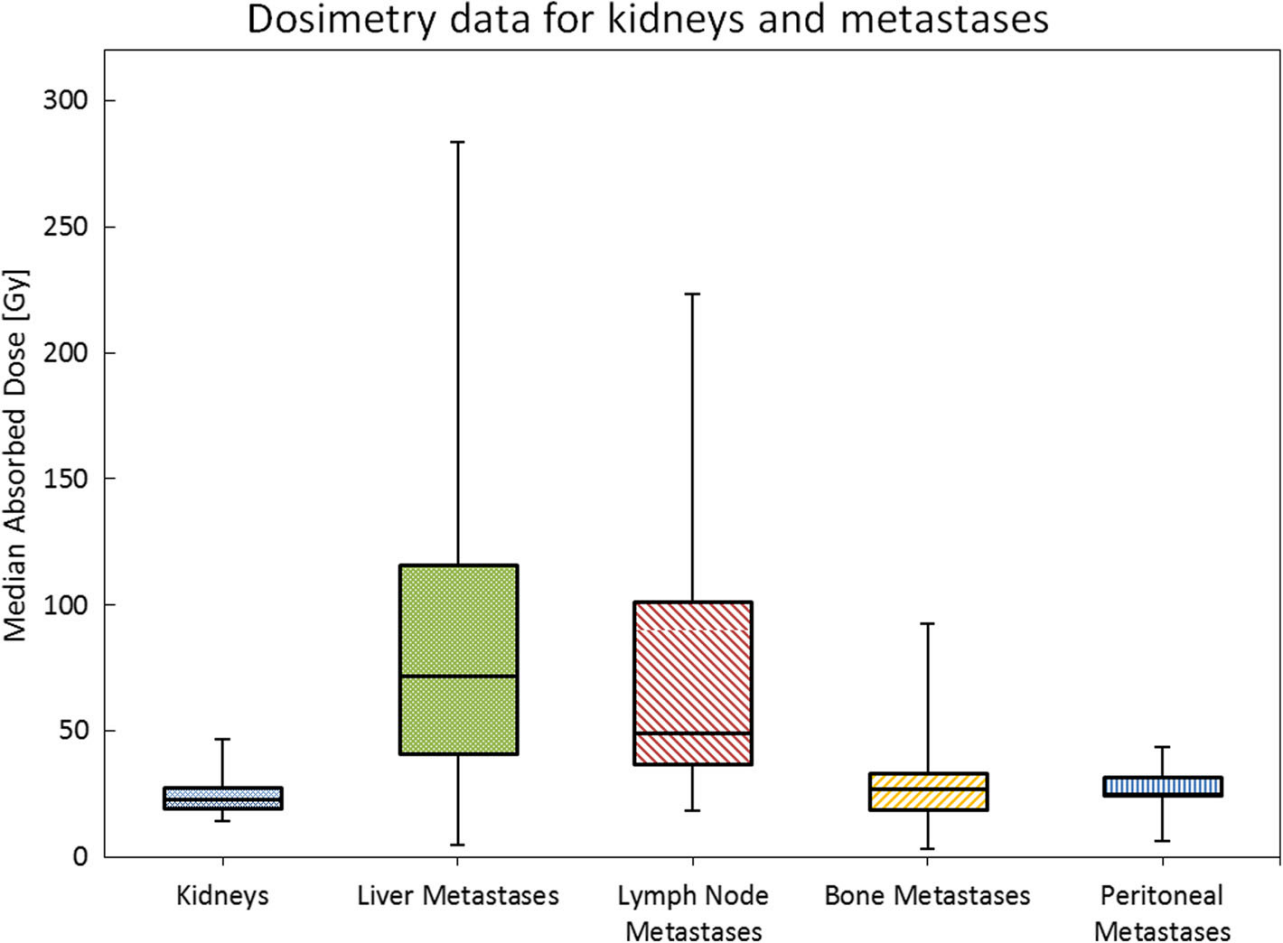
Cumulative absorbed Dose for kidneys and tumor lesions. Boxplot-Analysis of the cumulative dose for kidneys and tumor lesions. A significantly higher cumulative dose was found for liver metastases and lymph node metastases compared to bone and peritoneal tumor lesions each

Tumor dosimetry was performed in a total number of 204 tumor lesions including 152 liver metastases, 26 lymph node metastases, 21 bone metastases and 5 peritoneal metastases. The mean absorbed cumulative tumor dose for all tumor lesions was 76.4 ± 56.9 Gy with a mean absorbed dose of 2.30 ± 1.83 Gy per GBq of ^177^Lu-DOTATATE. The boxplots for cumulative doses among the kidneys and the different tumor sides are shown in Fig. 1. The mean cumulative dose in liver and lymph node metastases (84.9 ± 57.9 Gy and 73.9 ± 53.6 Gy, respectively) was significantly higher compared to bone or peritoneal metastases 30.0 ± 21.2 Gy and 26.3 ± 13.3 Gy, respectively; *p* < 0.01). The mean absorbed kidney dose per cycle was 4.0 ± 1.1 Gy (0.54 ± 0.15 Gy/GBq) with a mean cumulative dose of 23.8 ± 6.5 Gy. Pearson correlation revealed no significant correlation between cumulative absorbed renal dose and decrease of TER. Over the time of six cycles there was a slight, but statistically not significant increase of the mean absorbed kidney dose (+ 0.85 Gy between cycle 1 and 6) opposed to a decrease of absorbed dose in metastases (− 3.25 Gy between cycle 1 and 6). A strong inverse correlation of increasing mean kidney doses and decreasing mean tumor doses was detected (Spearman-Coefficient: − 0.9, *p* < 0.01).

### Efficacy and survival after salvage PRRT

**Fig. 2:**
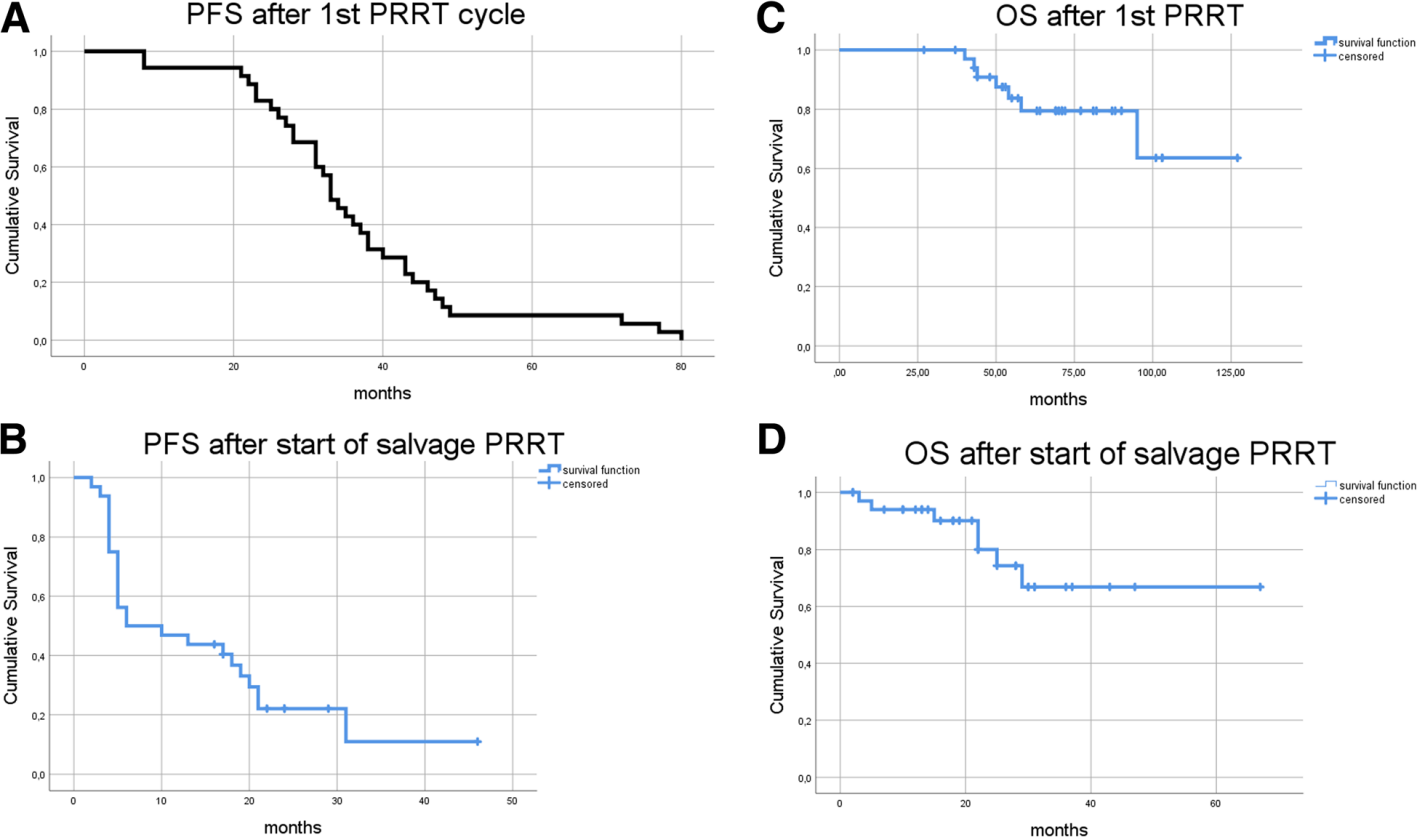
Kaplan-Meier-Survival analysis for PFS and OS after 1st and salvage PRRT. Kaplan-Meier-Survival Analysis for PFS and OS. Every patient showed initial response to four cycles of ^177^Lu-DOTATATE and later progression prior to salvage PRRT. Median PFS after the start of PRRT was 33 months (**a**). The median PFS after start of salvage therapy was 6 months (**b**) median follow-up time: 25 months; 3 patients lost to follow-up, 8 patients censored). Median OS was not reached during a median follow-up of 71 months from start of 1st PRRT cycle (**c**) and 25 months from the start of salvage PRRT (**d**)

Median follow-up after first PRRT was 71 months (95% CI: 64–78) and 25 months (95% CI: 18–31) after start of salvage PRRT. ^68^Ga-DOTATATE PET/CT, diagnostic CT and/or MRI based tumor for assessment of best response after salvage therapy was available for 32 patients. Three patients were lost to follow-up without PET-, CT or MRI-scans during follow-up and excluded from the analysis. In the remaining 32 patients one patient (3.1%) showed partial regression (PR), 26 patients (81.3%) showed stable disease (SD) and 5 patients (15.6%) progressive disease (PD) according to RECIST 1.1. Initial median PFS after the first cycle of PRRT was 33 months (95% CI: 30–36) (see Fig. 2). Median PFS after salvage therapy was 6 months (95% CI: 0–16; 8/32 patients censored). Median OS after start of first PRRT and start of salvage PRRT was not reached, mean OS was 105 months (95% CI: 92–119) and 51 months (95% CI: 41–61) after start of initial and salvage PRRT, respectively. Eight patients died from their cancer with a median of 50 and 22 months after start of PRRT and salvage PRRT, respectively. Hematological, clinical and dosimetry parameters such as WBC, erythrocytes, Hb, platelet count, cumulative absorbed kidney dose, Ki67, sex, post-therapeutical weight loss, therapy response and age had no significant impact on survival according to the univariate cox hazard model. However, there was a tendency towards higher overall survival in patients who received a mean cumulative absorbed kidney dose higher than 19.5 Gy (*p* = 0.052).

## Discussion

In this retrospective study the toxicity, dosimetry and efficacy of salvage PRRT using ^177^Lu-DOTATATE was analyzed in 35 metastasized and multimodally pretreated NET patients. Previously published data on salvage PRRT reported a tolerable toxicity with promising treatment efficacy [12, 14]. Nonetheless, hematological and renal toxicity are still regarded as the main side effects and dose limiting factors for PRRT [11] and are of particular concern in the salvage setting. Although we observed a significant decrease in WBC, erythrocytes and platelet count both after initial and salvage PRRT, blood values recovered and were still within the limits of current PRRT guidelines. Recently Löser et al. presented similar results with significantly decreasing platelets and WBC in 30 patients undergoing ^177^Lu-DOTATATE therapy with 17 and 13 patients receiving a cumulative therapy activity of ≤29.6 GBq (conforming to ≤4 PRRT cycles with 7.4 GBq) or > 29.6 GBq (conforming to salvage PRRT), respectively [13]. Interestingly, platelet counts of ≥399,000 cells/μl was associated with worse survival, which was not observed in our cohort. In our analysis one patient (2.9%) showed reversible grade 3 hematotoxicity after salvage PRRT (anemia with reduced hemoglobin to 6.8 g/dl). Studies with larger patient numbers describe similar rates of grade 3/4 hematotoxicity. Recently, Garske-Román et al. presented data on 200 patients receiving ^177^Lu-DOTATATE therapy according to a dosimetry based study protocol with the goal to not exceed a cumulative kidney dose of 23 Gy [25]. Grade 3 or 4 hematotoxicity was observed in 30 patients (15%). Bergsma et al. observed grade 3/4 hematotoxicity in 34 of 320 patients (11%) receiving up to 29.6 GBq of ^177^Lu-DOTATATE. Data on patients receiving ≥8 ^177^Lu-DOTATATE therapy cycles have been published recently by Yordanova et al. in 15 patients [15] and by van der Zwan et al. in 13 patients within a larger cohort of 181 salvage PRRT patients receiving ≥5 cycles [14]. Yordanova et al. observed grade 3/4 hematotoxicity in 4 patients (23%) between PRRT cycles 1 and 4, in 3 patients (13%) between cycles 5 and 8 and in none of 9 patients between cycles 9 to 13. Similarly van der Zwan et al. observed grade 3 /4 hematotoxicity in 13 patients (7.2%) after salvage therapy (cylces 5 and 6) and in 1 patient (7.7%) after re-salvage therapy (cycle 7 and 8). Regarding nephrotoxicity, no grade 3 / 4 toxicity was obversved in our cohort. The median annual decrease in TER of 0.03 ± 0.07 (2.25 ± 0.48%) normed to the lower limit is comparable to data by Werner et al. [23]. Contrary to their findings we could not detect a significant correlation between high initial median TER and a decrease in TER. In our cohort the decrease of absolute TER before initial and after salvage PRRT was higher than reported by Werner et al. indicating a preserved kidney function in their cohort. This might be explained by the larger number of therapy cycles in our salvage PRRT cohort. Kidney dosimetry revealed a mean cumulative absorbed dose of 23.8 ± 6.5 Gy in the salvage PRRT setting without correlation with loss of TER. This indicates that salvage PRRT with a median of 6 cycles results in an absorbed kidney dose which is within the limit of 23 Gy, established as the maximum tolerated dose by means of external beam radiation. In a large group of 323 NET patients Bergsma et al. observed no grade 3 or 4 nephrotoxicity or annual decrease of renal function > 20% and calculated a mean kidney dose of 20.1 ± 4.9 Gy in 228 patients by planar scintigraphy [26]. They concluded that the threshold of 23 Gy seems to be too low. Low rates of Grade 3 and 4 nephrotoxicity even in the salvage setting support this conclusion [14, 15]. However, as the kidneys are still considered as the dose limiting organs, SPECT based dosimetry protocols as presented by Garske-Román et al. represent interesting approaches, particularly as tumor based dosimetry is not feasible in NET [25]. In contrast to other authors we found a significant difference in cumulative absorbed doses of different locations of metastases with higher doses in liver and lymph node metastases compared to bone and peritoneal lesions [13]. Supporting the thesis of a tumor sink effect in NET [27], we found a significant inverse correlation of increasing mean kidney doses and decreasing mean tumor doses over the course of initial and salvage PRRT. This supports the thesis that lower mean absorbed tumor doses lead to higher mean absorbed kidney doses. However, the high number of other therapy options including liver targeted therapies might also have an impact on SSTR expression and absorbed dose in tumors. Further trials with higher patient numbers and stratification according to previous therapies are needed to answer this question.

Different response assessment criteria have been applied for therapy monitoring in NET patients after PRRT. RECIST 1.1 has been validated for NET patients within the NETTER-1 trial [9]. Nonetheless, response data specifically for the salvage PRRT setting are reported relatively rarely. Compared to other studies our response rates seem considerably lower. A comparative presentation is given in Table 5. This might be explained by the multimodal treatment of NET patients in our center, which is based on an interdisciplinary tumor board decision. Salvage PRRT patients included in the cohorts of Sabet et al. and van der Zwan et al. did not receive additional therapies prior to salvage PRRT. For instance, in our cohort a considerably high number of patients received local ablative liver therapies not only prior to the first PRRT (34.4%) but additionally prior to salvage PRRT (25.7%). Other intermediate therapies included surgery, bone targeted therapy, everolimus and chemotherapy. Thus salvage PRRT was not the first therapy option at the time of progression after initial PRRT but third or fourth line in many cases. Median PFS after salvage PRRT at a median follow-up of 25 months was only 6 months with 8 patients being censored at the time of analysis. Median PFS after four initial PRRT cycles was 33 months and within the range of 27–40 months as described in other studies [7, 8, 25]. This could again be explained by the relatively late sequence of salvage PRRT in a considerably high number of patients in our cohort. Other groups report median PFS after salvage PRRT ranging from 13 to 18.9 months [12, 14, 15]. The only difference compared to these studies is the multimodal treatment between initial PRRT and salvage PRRT in our patient cohort. Garske-Román et al. present a similar patient cohort to ours [25]. However the therapy sequence is not further specified and local ablative liver therapies were not included. Additionally the PFS has been described for the whole patient cohort with respect to the cumulative kidney dose and not the number of therapy cycles. Thus the PFS of salvage PRRT patients is not described. However, in 39 patients receiving 5 or more cycles of ^177^Lu-DOTATATE, a cumulative dose of 23 Gy to the kidneys was reached only in 26 patients and 19 had died. However, they defined the common threshold of 23 Gy to be associated with a higher survival regardless of the number of therapy cycles [25]. In our cohort univariate Cox Hazard analysis revealed a tendency towards higher overall survival in patients with a mean cumulative absorbed kidney dose higher than 19.5 Gy (*p* = 0.052) compared to patients who received lower doses. As PRRT is mainly performed with a standardized dose of 7.4 GBq per cycle the added value of individualized dosimetry is still a matter of debate. Our study and the data by Garske-Román et al. show that higher absorbed kidney dose are associated with higher survival and remission rates. Personalized dosimetry enables to aim for the maximum of the tolerable dose to receive the best possible outcome.

**Table 5:** Best response after salvage PRRT

Authors	patients n=	Number of PRRT cycles	Median Follow-Up [months]	CR/PR/MR^a^	SD^b^	PD^c^
This study	35	5–8	25	1 (3.1)	26 (81.6)	5 (15.3)
Sabet et al. [12]	33	5–8	23	7 (21.2)	14 (42.4)	11 (33.3)
van der Zwan et al. [14]	168	5–8	30.4	26 (15.5)	100 (59.5)	33 (19.6)

^a^*CR/PR/MR* Complete/Partial/Minor Response, ^b^*SD* Stable Disease, ^c^*PD* Progressive Disease

Data is presented as number (%) of patients. Assessment of best response was based on RECIST 1.1 in the current study and in [14] and on SWOG criteria in [12]; 3 and 9 patients were excluded in the current study and in [14], respectively

One limitation of this study is the retrospective, monocentric study design. The patient cohort is relatively small and although the heterogeneity of different treatment options during the course of disease appears to have a major impact on response and survival, the interpretation is limited. Furthermore patients in our cohort present with various NET primary tumors with different tumor characteristics in terms of agressiveness and prognosis. This particularly applies for lung NET with typical and atypical histological variants, which have high impact on therapy response and surivival. Due to the small sample size, a subgroup analysis was performed only in small intestinal NET and demonstrated longer PFS compared to other primary tumors (data not shown). However, such data needs validation in larger cohorts, such as recently described by van der Zwan et al. [14]. Nonetheless it could be demonstrated that the majority of our heterogeneous patient population shows disease stabilization in terms of PR and SD after multimodal therapy and progression.

## Conclusion

Salvage PRRT using ^177^Lu-DOTATATE is feasible and safe. Despite a relatively short median PFS, salvage PRRT was still an effective therapy option in extensively pretreated, progressive NET patients. A cumulative absorbed kidney dose higher than 19.5 Gy was associated with a tendency towards a longer overall survival confirming the value of personalized dosimetry for PRRT.

## Additional file

Additional file 1: **Table S5.** Mean TER changes after PRRT in all patients (*n*=35) (DOCX 14 kb)

## Data Availability

The datasets supporting the conclusions of this article are included within the tables (Tables 1, 2, 3, 4, 5, Additional file 1: Table S5, Figs. 1, 2). All measurements were collected and recorded in Microsoft Excel. All materials will be made available upon reasonable request by the corresponding author.

## Abbreviations

^177^Lu-DOTATATE: ^177^Lu-DOTA-D-Phe-Tyr3-octreotate
^68^Ga-DOTATATE: ^68^Ga-DOTA-D-Phe-Tyr3-octreotate
CR: Complete response
CTCAE: Common Terminology Criteria for Adverse Events
EMA: European Medicines Agency
FDA: Food and Drug Administration
NET: Neuroendocrine tumor
PD: Progressive disease
PET/CT: Positron emission tomography/computed tomography
PFS: Progression free survival
PR: Partial response
PRRT: Peptide receptor radionuclide therapy
PS: Overall survival
RECIST 1.1: Response evaluation criteria in solid tumors version 1.1
ROC: Receiver operating characteristics
SD: Stable disease
SD: Standard deviation
SEM: Standard error of the mean
SPECT: Singe photon emission computed tomography
SSTR: Somatostatin receptor
TER: Tubular excretion rate
WBC: White blood cells

## References

[1] Dasari A, Shen C, Halperin D, Zhao B, Zhou S, Xu Y, Shih T, Yao JC (2017). Trends in the incidence, prevalence, and survival outcomes in patients with neuroendocrine tumors in the United States. JAMA Oncol.

[2] Man D, Wu J, Shen Z, Zhu X (2018). Prognosis of patients with neuroendocrine tumor: a SEER database analysis. Cancer Manag Res.

[3] Riihimaki M, Hemminki A, Sundquist K, Sundquist J, Hemminki K (2016). The epidemiology of metastases in neuroendocrine tumors. Int J Cancer.

[4] Dromain C, Pavel ME, Ruszniewski P, Langley A, Massien C, Baudin E, Caplin ME, Group CS (2019). Tumor growth rate as a metric of progression, response, and prognosis in pancreatic and intestinal neuroendocrine tumors. BMC Cancer.

[5] Reubi JC (2004). Somatostatin and other peptide receptors as tools for tumor diagnosis and treatment. Neuroendocrinology.

[6] Kwekkeboom DJ, Teunissen JJ, Bakker WH, Kooij PP, de Herder WW, Feelders RA, van Eijck CH, Esser JP, Kam BL, Krenning EP (2005). Radiolabeled somatostatin analog [177Lu-DOTA0,Tyr3] octreotate in patients with endocrine gastroenteropancreatic tumors. J Clin Oncol.

[7] Kwekkeboom DJ, de Herder WW, Kam BL, van Eijck CH, van Essen M, Kooij PP, Feelders RA, van Aken MO, Krenning EP (2008). Treatment with the radiolabeled somatostatin analog [177 Lu-DOTA 0,Tyr3]octreotate: toxicity, efficacy, and survival. J Clin Oncol.

[8] Ezziddin S, Attassi M, Yong-Hing CJ, Ahmadzadehfar H, Willinek W, Grunwald F, Guhlke S, Biersack HJ, Sabet A (2014). Predictors of long-term outcome in patients with well-differentiated gastroenteropancreatic neuroendocrine tumors after peptide receptor radionuclide therapy with 177Lu-octreotate. J Nucl Med.

[9] Strosberg J, El-Haddad G, Wolin E, Hendifar A, Yao J, Chasen B, Mittra E, Kunz PL, Kulke MH, Jacene H (2017). Phase 3 trial of (177)Lu-Dotatate for midgut neuroendocrine tumors. N Engl J Med.

[10] Pape UF, Niederle B, Costa F, Gross D, Kelestimur F, Kianmanesh R, Knigge U, Oberg K, Pavel M, Perren A (2016). ENETS consensus guidelines for neuroendocrine neoplasms of the appendix (excluding goblet cell carcinomas). Neuroendocrinology.

[11] Bodei L, Mueller-Brand J, Baum RP, Pavel ME, Horsch D, O'Dorisio MS, O'Dorisio TM, Howe JR, Cremonesi M, Kwekkeboom DJ (2013). The joint IAEA, EANM, and SNMMI practical guidance on peptide receptor radionuclide therapy (PRRNT) in neuroendocrine tumours. Eur J Nucl Med Mol Imaging.

[12] Sabet A, Haslerud T, Pape UF, Sabet A, Ahmadzadehfar H, Grunwald F, Guhlke S, Biersack HJ, Ezziddin S (2014). Outcome and toxicity of salvage therapy with 177Lu-octreotate in patients with metastatic gastroenteropancreatic neuroendocrine tumours. Eur J Nucl Med Mol Imaging.

[13] Loser A, Schwarzenbock SM, Heuschkel M, Willenberg HS, Krause BJ, Kurth J (2018). Peptide receptor radionuclide therapy with 177Lu-DOTA-octreotate: dosimetry, nephrotoxicity, and the effect of hematological toxicity on survival. Nucl Med Commun.

[14] van der Zwan WA, Brabander T, Kam BLR, Teunissen JJM, Feelders RA, Hofland J, Krenning EP, de Herder WW. Salvage peptide receptor radionuclide therapy with [(177)Lu-DOTA,Tyr(3)]octreotate in patients with bronchial and gastroenteropancreatic neuroendocrine tumours. Eur J Nucl Med Mol Imaging. 2018. 10.1007/s00259-018-4158-1 PMC6351514 30267116

[15] Yordanova A, Mayer K, Brossart P, Gonzalez-Carmona MA, Strassburg CP, Essler M, Ahmadzadehfar H (2017). Safety of multiple repeated cycles of (177)Lu-octreotate in patients with recurrent neuroendocrine tumour. Eur J Nucl Med Mol Imaging.

[16] Ilhan H, Wang H, Gildehaus FJ, Wangler C, Herrler T, Todica A, Schlichtiger J, Cumming P, Bartenstein P, Hacker M (2016). Nephroprotective effects of enalapril after [177Lu]-DOTATATE therapy using serial renal scintigraphies in a murine model of radiation-induced nephropathy. EJNMMI Res.

[17] Delker A, Ilhan H, Zach C, Brosch J, Gildehaus FJ, Lehner S, Bartenstein P, Boning G (2015). The influence of early measurements onto the estimated kidney dose in [(177)Lu][DOTA(0),Tyr(3)]Octreotate peptide receptor radiotherapy of neuroendocrine tumors. Mol Imaging Biol.

[18] Delker A, Fendler WP, Kratochwil C, Brunegraf A, Gosewisch A, Gildehaus FJ, Tritschler S, Stief CG, Kopka K, Haberkorn U (2016). Dosimetry for (177)Lu-DKFZ-PSMA-617: a new radiopharmaceutical for the treatment of metastatic prostate cancer. Eur J Nucl Med Mol Imaging.

[19] Gosewisch A, Delker A, Tattenberg S, Ilhan H, Todica A, Brosch J, Vomacka L, Brunegraf A, Gildehaus FJ, Ziegler S (2018). Patient-specific image-based bone marrow dosimetry in Lu-177-[DOTA(0),Tyr(3)]-Octreotate and Lu-177-DKFZ-PSMA-617 therapy: investigation of a new hybrid image approach. EJNMMI Res.

[20] Garske U, Sandstrom M, Johansson S, Sundin A, Granberg D, Eriksson B, Lundqvist H (2012). Minor changes in effective half-life during fractionated 177Lu-octreotate therapy. Acta Oncol.

[21] Bolch WE, Eckerman KF, Sgouros G, Thomas SR (2009). MIRD pamphlet no. 21: a generalized schema for radiopharmaceutical dosimetry--standardization of nomenclature. J Nucl Med.

[22] Werner RA, Bluemel C, Lapa C, Muegge DO, Kudlich T, Buck AK, Herrmann K (2014). Pretherapeutic estimation of kidney function in patients treated with peptide receptor radionuclide therapy: can renal scintigraphy be safely omitted?. Nucl Med Commun.

[23] Werner RA, Beykan S, Higuchi T, Luckerath K, Weich A, Scheurlen M, Bluemel C, Herrmann K, Buck AK, Lassmann M (2016). The impact of 177Lu-octreotide therapy on 99mTc-MAG3 clearance is not predictive for late nephropathy. Oncotarget.

[24] Eisenhauer EA, Therasse P, Bogaerts J, Schwartz LH, Sargent D, Ford R, Dancey J, Arbuck S, Gwyther S, Mooney M (2009). New response evaluation criteria in solid tumours: revised RECIST guideline (version 1.1). Eur J Cancer.

[25] Garske-Roman U, Sandstrom M, Fross Baron K, Lundin L, Hellman P, Welin S, Johansson S, Khan T, Lundqvist H, Eriksson B (2018). Prospective observational study of (177)Lu-DOTA-octreotate therapy in 200 patients with advanced metastasized neuroendocrine tumours (NETs): feasibility and impact of a dosimetry-guided study protocol on outcome and toxicity. Eur J Nucl Med Mol Imaging.

[26] Bergsma H, Konijnenberg MW, van der Zwan WA, Kam BL, Teunissen JJ, Kooij PP, Mauff KA, Krenning EP, Kwekkeboom DJ (2016). Nephrotoxicity after PRRT with (177)Lu-DOTA-octreotate. Eur J Nucl Med Mol Imaging.

[27] Beauregard JM, Hofman MS, Kong G, Hicks RJ (2012). The tumour sink effect on the biodistribution of 68Ga-DOTA-octreotate: implications for peptide receptor radionuclide therapy. Eur J Nucl Med Mol Imaging.

