# Supplementary material for: Salvage PRRT with 177Lu-DOTA-octreotate in extensively pretreated patients with metastatic neuroendocrine tumor (NET): dosimetry, toxicity, efficacy, and survival
Source: BMC Cancer. 2019 Aug 8;19:788. doi: 10.1186/s12885-019-6000-y (PMC6686531; doi:10.1186/s12885-019-6000-y)
Supplement: Supplementary file 1 — Table S5. Mean TER changes after PRRT in all patients (n=35) (DOCX 14 kb) [file 12885_2019_6000_MOESM1_ESM.docx]

**Additional file 1: Table S1 Mean TER changes after PRRT in all patients (n=35)**

|  | Before PRRT | After 4 PRRT | After salvage PRRT |
| --- | --- | --- | --- |
| Mean TER (ml/min/1.73 m²) | 226 | 207 | 191 |
| Mean TER Low Boundary (ml/min/1.73 m²) | 172 | 169 | 164 |
| Mean TER/Low Boundary | 1.31 | 1.23 | 1.17 |
| Mean decrease TER p.a. (SD) (ml/min/1.73 m²) |  |  | -8.337 (± 12.4) |
| Mean decrease normed TER p.a. (SD) (ml/min/1.73 m²) |  |  | -0.03 (± 0,07) |
| Mean decrease normed TER p.a. % (SD) (ml/min/1.73 m²) |  |  | -0.03 (± 0,05) |
|  |  |  |  |
